# Cost-effectiveness of a victim improvement package: randomised controlled trial for reduction of continued symptoms of depression or anxiety in older victims of community crime

**DOI:** 10.1192/bjo.2025.10937

**Published:** 2026-01-07

**Authors:** Monica Panca, Marc Serfaty, Jessica Satchell, Rachael Maree Hunter

**Affiliations:** Comprehensive Clinical Trials Unit, University College London, London, UK; Department of Epidemiology and Applied Clinical Research, Division of Psychiatry, University College London, London, UK; The Priory Hospital North London, London, UK; Priment Clinical Trials Unit, University College London, London, UK

**Keywords:** Community crime, older victims, depression, anxiety, cost-utility

## Abstract

**Background:**

Crime has significant impact on older victims. High rates of anxiety and depression may be associated with crimes.

**Aims:**

This paper aims to evaluate the cost-effectiveness of a victim improvement package (VIP) for the reduction of continued symptoms of depression or anxiety in older victims of community crime, from the societal perspective, in a three-step, parallel-group, single-blind randomised controlled trial.

**Method:**

Participants (*N* = 131) were randomised to receive either the VIP intervention in addition to treatment as usual (TAU) (*n* = 65), or to TAU alone (*n* = 66). Service resource use was collected using the Client Service Receipt Inventory and health-related quality-of-life data via the EQ-5D-5L instrument at 3 months post-crime (baseline), 6 months post-crime (post-intervention) and 9 months post-crime (follow-up).

**Results:**

The mean cost of the VIP intervention was estimated at £1330 per participant in the intervention arm. The mean difference in costs between the VIP and TAU arms over the 6-month trial duration was −£881 (95% CI: −£5947 to £4186). The mean difference in quality-adjusted life-years (QALYs) was −0.011 (95% CI: −0.042 to 0.020).

**Conclusions:**

The addition of VIP to TAU for older victims of community crime generated a lower mean point estimate for costs, and failed to improve quality of life compared with TAU alone. While this places VIP in the south-west quadrant of the cost-effectiveness plane, the magnitude and significance of the QALY difference do not justify declaring VIP cost-effective or TAU not cost-effective. Future research is needed to identify the most cost-effective intervention.

Crime is a major public health problem that results in serious health consequences for its victims, including increased risk of morbidity, mortality, institutionalisation and hospital admission, and has a negative effect on both families and society.^1^ Data from the Office for National Statistics Census 2021 showed that about 21.1% of adults who were victims of any crime, excluding fraud and computer misuse, reported symptoms of depression in 2021 compared with 12.0% of those who were not victims. Older people may be specifically targeted for certain crimes because of their age, and some may be particularly vulnerable due to ill-health, dementia or social isolation.^2,3^ Crime has significant emotional, psychological, physical, financial and social impact on older victims, with high rates of anxiety and depression.^4^ Both anxiety and depression contribute to increased societal costs through higher health care utilisation.^5,6^ Older people, in particular, tend to be high utilisers of medical services, and service demands increase with clinical depression.^7^ Psychological therapies have been demonstrated to be as effective as antidepressant medication,^8^ with one meta-analysis showing a patient preference for psychological over pharmacological treatment^9^ in anxiety and depression. Psychological therapies such as cognitive behavioural therapy (CBT) have been shown to be both clinically and cost-effective,^10,11^ and have demonstrated effectiveness in treating, maintaining progress and preventing relapse in both depression and anxiety disorders.^12–15^ The victim improvement package (VIP) study suggested that, despite being a victim of crime, less than 13% of older people signposted to their GP acted on this and less than a third of these were offered any treatment.^16^

The aim of the economic evaluation reported here was to assess the cost-effectiveness of VIP intervention for the reduction of continued symptoms of depression and/or anxiety in older victims of community crime.

## Method

### Trial design and population

The study was conducted across 12 local authority areas. Recruitment commenced in May 2017 and was extended on three occasions to the end of September 2022. The first participant was randomised on 20 November 2017 and the final participant, final follow-up was on 16 February 2023. Police-reported victims of community crime aged 65 years and over, with continued symptoms of anxiety and/or depression, were recruited into a parallel-group, single-blind, individually randomised controlled trial (RCT) of a CBT-informed VIP added to treatment as usual (TAU) compared with TAU alone.

The study included community crime, as defined by the World Health Organization,^17^ extended to all crimes (e.g. assault, burglary, fraud) but excluded domestic and sexual violence. A detailed description of the protocol has previously been published.^18^ Pressures on staff resourcing, the COVID-19 pandemic and possibly poorer public confidence in the police have impacted on recruitment and retention of participants, and therefore the trial was underpowered.

The trial consisted of three steps. Step 1: police community support officers screened participants for depressive symptoms using Patient Health Questionnaire 2 (PHQ-2; >3)^19^ and/or anxiety on the Generalized Anxiety Disorder 2-item (GAD-2; ≥2)^20^ within 2 months of reporting a community crime, and collected demographic information and consent to data-share. Step 2: participants were re-screened at 3 months using the same measures by researchers and, if participants were still distressed at step 3, they were assessed for eligibility and consented into the RCT.

Participants allocated to the VIP intervention were offered up to 10 sessions with a therapist from a mental health charity over a period of 3 months. The VIP manual, available from the chief investigator^21^ and developed and piloted in a RCT (Helping Aged Victims of Crime study),^16^ served as a guide and followed the following format. Session 1: a narrative of the crime, underlying beliefs and behaviours and how these have changed; session 2: psychoeducation about crime and an introduction to CBT; sessions 3–8: mood diaries to identify unhelpful thinking and behaviours; guided discovery to challenge beliefs about crime, personal vulnerability and safety; and behavioural experiments to challenge unhealthy avoidances; sessions 9–10: relapse prevention. Within the constraints of the trial and the number of sessions available, the participant and therapist collaboratively agreed when the therapy should terminate. All participants received usual care as managed by their GP.

Participants allocated to the TAU arm continued their routine care, which could include referral/self-referral to private therapy or Improving Access to Psychological Therapies.

### Ethics

The authors assert that all procedures contributing to this work comply with the ethical standards of the relevant national and institutional committees on human experimentation, and with the Helsinki Declaration of 1975 as revised in 2013. The project was approved by the UCL Ethics Committee (project no. 6960/001). Signed informed consent was obtained for all participants. The International Standard Randomised Controlled Trial number is ISRCTN16929670 (https://doi.org/10.1186/ISRCTN16929670).

### Overview

The economic evaluation was conducted using individual participant cost-and-effect data collected alongside the VIP trial, and was performed from a broader societal perspective, including health and social, legal and criminal justice resource use, out-of-pocket expenses and informal care. The time horizon of the analysis was the duration of the trial, with assessments of costs and outcomes at the following time points: 3 months post-crime (baseline), 6 months post-crime (post-intervention) and 9 months post-crime (follow-up). The primary analysis was based on available data and conducted according to the intention-to-treat (ITT) principle.^18^ The report of this health economic evaluation followed Consolidated Health Economics Evaluation Reporting Standards guidance.^22^

### Service utilisation

An adapted version of the Client Service Receipt Inventory (CSRI)^23^ was used to measure individual-level service resource use. It was also utilised to record all criminal justice services (UK police force, legal/non-legal services, victim support); social and legal advice services (social worker, home care help by local authority, housing support, advice agencies, advice lines, charity services); healthcare services (in-patient, out-patient, accident and emergency, ambulance/paramedics, day/rehabilitation centre, primary care and community services, non-trial psychology services); informal care; and out-of-pocket expenses. Information on medications used included drug name, dose and duration of administration.

### Costs

All costs are reported in 2021–2022 UK pounds (£), adjusted for inflation where necessary, using the National Health Service (NHS) Cost Inflation Index published by the Personal Social Services Research Unit (PSSRU 2022), University of Kent, Canterbury. Discounting was not applied given that the duration of follow-up did not exceed 12 months.

Health and social care resource use was costed using unit costs from those of health and social care (PSSRU 2022) and NHS reference costs published by the Department of Health (2021–2022) (Supplementary Table 1 available at https://doi.org/10.1192/bjo.2025.10937). The costs of medications were estimated from the British National Formulary (bnf.nice.org.uk; Supplementary Table 2). Informal caregivers were costed at the rate of paid caregivers based on the assumption that, in the absence of an informal caregiver, a paid carer would be required to undertake the same role.^24^ An hour of unpaid care was costed as the median hourly wage of a home care worker (Supplementary Table 1).

The wider costs of service use, collected using CSRI, included those of the criminal justice service and social and legal advice services, and were calculated using ‘The economic and social costs of crime’^1^ published by the UK Home Office in 2018 (Supplementary Table 1). Data on state benefits received by participants were also recorded.

### VIP intervention costs

A micro-costing approach (a cost estimation method that involves direct enumeration of the cost of each resource required)^25^ was adopted to estimate the additional resource use and costs associated with the VIP intervention. Study records of the number of therapists attending training sessions were used to track resources utilised in the delivery of the training programmes, including trainee and trainer time, to calculate the fixed cost of training. For the delivery of the intervention, the number of sessions delivered and the time each therapist spent with a participant were recorded. Unit costs for therapists to train for, and deliver, the intervention were based on per-hour of direct contact in 2016–2017 (PSSRU 2017). An annual inflator was used to uprate the costs to 2021/2022 values (The Personal Social Services (PSS) Pay & Prices Index, PSSRU 2022).

### Outcomes

The primary health outcome measure used in the economic evaluation was quality-adjusted life-years (QALYs), calculated from participant responses to the EQ-5D 5-level (EQ-5D-5L) (www.euroqol.org) instrument. QALY is the reference case outcome recommended by the National Institute for Health and Care Excellence (NICE) for use in economic evaluations.^26^ EQ-5D-5L is a generic health-related measure of quality of life that contains five domains: mobility, self-care, usual activities, pain/discomfort and anxiety/depression, each of which has five levels. Each of the five items is rated on a five-point scale, from no problem to extreme problems. The self-completed questionnaire captured participants’ perspectives on health status. The cross-walk algorithm,^27^ which maps EQ-5D-5L value sets to the currently available three-level version of EQ-5D-3L, was used.^28^

### Cost-utility analysis

Analyses were prespecified in the economic evaluation section of the statistical analysis,^18^ signed off prior to database lock. The analysis was performed in Stata version 18 for Windows (www.stata.com).

Descriptive statistics for the number of participants using a type of contact, and mean number of contacts for participants who used that service, as collected by the CSRI, were reported at baseline (3 months post-crime), at the 6- and 9-month follow-up time points and over the 6-month duration of the trial.

Information collected on therapist training and time spent delivering the intervention, multiplied by the cost of the therapist for each participant, was used to calculate the patient-level cost of VIP intervention.

Mean cost per participant for the VIP intervention versus TAU was reported by type of service. Mean utility values at each time point and mean QALYs were also reported. Mean differences in cost and outcome were calculated using a mixed-effect model, with therapist clustering as a random effect, site as a fixed effect and adjustment for baseline cost/utilities. A bias-corrected and accelerated bootstrap method with 1000 replications was used to test for differences between the arms for costs and QALYs. Bootstrapped mean differences and their associated 95% confidence intervals are reported. Mean costs and QALYs were used to calculate the mean incremental cost per QALY gained from VIP intervention compared with TAU.

Estimates of bootstrapped mean cost and effectiveness were used to estimate the incremental cost-effectiveness ratio (ICER). The ICER for each replication was calculated by dividing the difference in total costs (incremental cost) by that in total health outcome (incremental effect) to provide a ratio of extra cost per extra unit of health effect. Uncertainty around cost-effectiveness outcomes was modelled by plotting bootstrapped results for incremental costs and outcomes on cost-effectiveness planes (CEPs).^29^ Cost-effectiveness acceptability curves (CEACs)^30^ were also constructed, using the bootstrap data from a range of values of willingness-to-pay (WTP) for a QALY gained. In line with NICE guidance,^26^ the probability that the VIP intervention is cost-effective compared with TAU at a WTP for a QALY gained of £20 000–£30 000 was reported.

### Missing data

Given the reduced sample size and potential for missing data, we looked at different methods to assess the implications of missing data. Multiple imputation was performed for missing values of utilities and costs using predictive mean matching (*k* = 50), which corresponds to the proportion of missing data (50%) and chained equations^31,32^ as a sensitivity analysis.

The imputation model included predictors of missingness, sociodemographic baseline data, follow-up scores, site and therapist as variables to impute the costs and utilities. Total costs, QALYs, ICERs, CEACs and CEPs were estimated using the imputed data and the method set out by Leurent et al.^33^

Participants were excluded from the multiple imputation analysis if they had no EQ-5D-5L or CSRI entries at baseline. Mixed-effects logistic regression was used to calculate baseline-adjusted differences in costs and outcomes and, subsequently, incremental costs and incremental outcomes. We ran the analytic model within each of the imputed data-sets using Rubin’s rules^34^ to control for variability among imputations. Bootstrapping was used only in conjunction with multiple imputation, to display uncertainty in the form of CEPs and CEACs.

### Sensitivity analysis

There were 23 (35%) participants allocated to the VIP arm who did not receive the intervention due to multiple causes: were not contactable (*n* = 2), withdrew from the study (*n* = 3), unable to leave house (*n* = 3) or in hospital (*n* = 1); there were also therapist-related capacity reasons (*n* = 5) and unknown reasons (*n* = 9). To complement the usual ITT estimates, a structural sensitivity analysis consisting of a per-protocol approach^35^ was conducted.

## Results

A total of 131 participants were randomised to the trial arms (65 to TAU plus VIP and 66 to TAU only). Participants appeared well balanced across trial arms with respect to demographic and other characteristics. Mean age (standard deviation) of participants was 72.1 (5.9) years in VIP and 72.1 (11.2) years in the TAU arm. Two-thirds of participants were female, and around a third were from ethnic minorities. Full demographic and clinical characteristics are presented in Supplementary Table 3.

### Service utilisation

Resource use by intervention arms is reported in Supplementary Table 4. Use of resources appeared similar for both intervention arms across all time points. However, contacts with primary care services for psychological reasons were significantly lower in the VIP intervention arm (mean difference −0.25 (95% CI: −0.48 to −0.02), *P* = 0.032) over 6 months. Although the numbers of those who used social and legal advice services were similar in both arms, the mean number (standard deviation) of services used was considerably higher in the TAU arm at all follow-up time points (31.75 (65.17) *v*. 9.63 (3.62) at 6-month follow-up; and 21.33 (41.11) *v*. 8.93 (7.68) at 9-month follow-up). In the same way, the number of those who had an informal carer was similar in both arms, although the mean number (standard deviation) of hours during which carers looked after participants in the TAU arm was higher than in the VIP arm (30.30 (50.06) *v*. 21.50 (8.38) at 6-month follow-up; and 49.67 (60.26) *v*. 12.71 (12.82) at 9-month follow-up).

### Costs

#### VIP intervention costs

A total of 49 therapists completed a 1-day training session in the VIP intervention (adapted from CBT) across 10 training days (7 h per session). Because of breaks between screening, four therapists were required to complete a refresher training day. Therapists delivered 315 VIP sessions (mean duration 1 h per session), with fortnightly supervision by the chief investigator via Zoom. The costs of the intervention included those of training the 49 therapists (plus 4 refresher sessions), delivering the intervention and consumables used during the face-to-face training sessions.

The total cost of the VIP intervention was estimated at £86 463. Given that 65 participants were allocated to the VIP arm to receive the intervention, this translated to a conservative estimate of £1330 per VIP participant in the intervention arm (Table 1). Sixty-one per cent of the total costs related to training of the therapists to help them adapt their skills for use in older victims of crime.

**Table 1:** Estimated costs of victim improvement package (VIP) intervention

Description	Cost
Training cost	
Band 9 (*n* = 1) psychologist delivered 10 (7 face-to-face/3 remote) training sessions of 7 h (+ 4 refresher training) for 49 therapists	£14 308
Therapists’ (*n* = 49) training	£35 672
Therapists’ (*n* = 4) refresher training	£2912
Total training costs	£52 892
Session delivery	
Sessions delivered (mean time per session, 1 h)	£32 760
Total session delivery costs	£32 760
Other costs	
Catering	£811
Total other cost	£811
Total cost	£86 463
Cost per participant in adapted VIP arm	£1330

#### Service utilisation costs

The services contributing to the costs in each arm are summarised in Supplementary Table 5. There were significantly lower costs for social and legal advice services (mean difference −£496.81 (95% CI: −£884.19 to −£109.44), *P* = 0.012) and the support provided by unpaid (informal) carers (mean difference −£6,643.49 (95% CI: −£13,058.54 to −£228.43), *P* = 0.042) over 6 months in the VIP arm compared with the TAU arm.

### Outcomes

Supplementary Table 6 reports the mean utility values per participant at each follow-up point. Differences in utility values between arms were not statistically significant at either 6-month follow up (mean difference VIP *v*. TAU −0.052, *P* = 0.493) or 9-month follow up (mean difference VIP *v*. TAU 0.043, *P* = 0.613).

### Cost-utility analyses

Table 2 reports the results of the cost-utility analysis. Participants in the VIP arm had a lower mean point estimate for costs (−£881 (95% CI: −£5947 to £4186)) and a negative mean point estimate for QALYs (−0.011 (95% CI: −0.042 to 0.022)) over the 6-month trial duration.

**Table 2:** Cost-effectiveness of victim improvement package intervention versus treatment as usual

Investigation used	Incremental cost (95% CI)	QALY gained (95% CI)	ICER	Probability cost-effectiveness £20 000	Probability cost-effectiveness £30 000
Complete case analysis (ITT analysis)	−£881 (−£5947 to £4186)	−0.011 (−0.042 to 0.020)	£80 167	36%	36%

QALY, quality-adjusted life-year; ICER, incremental cost-effectiveness ratio; ITT, intention-to-treat.

**Fig. 1:**
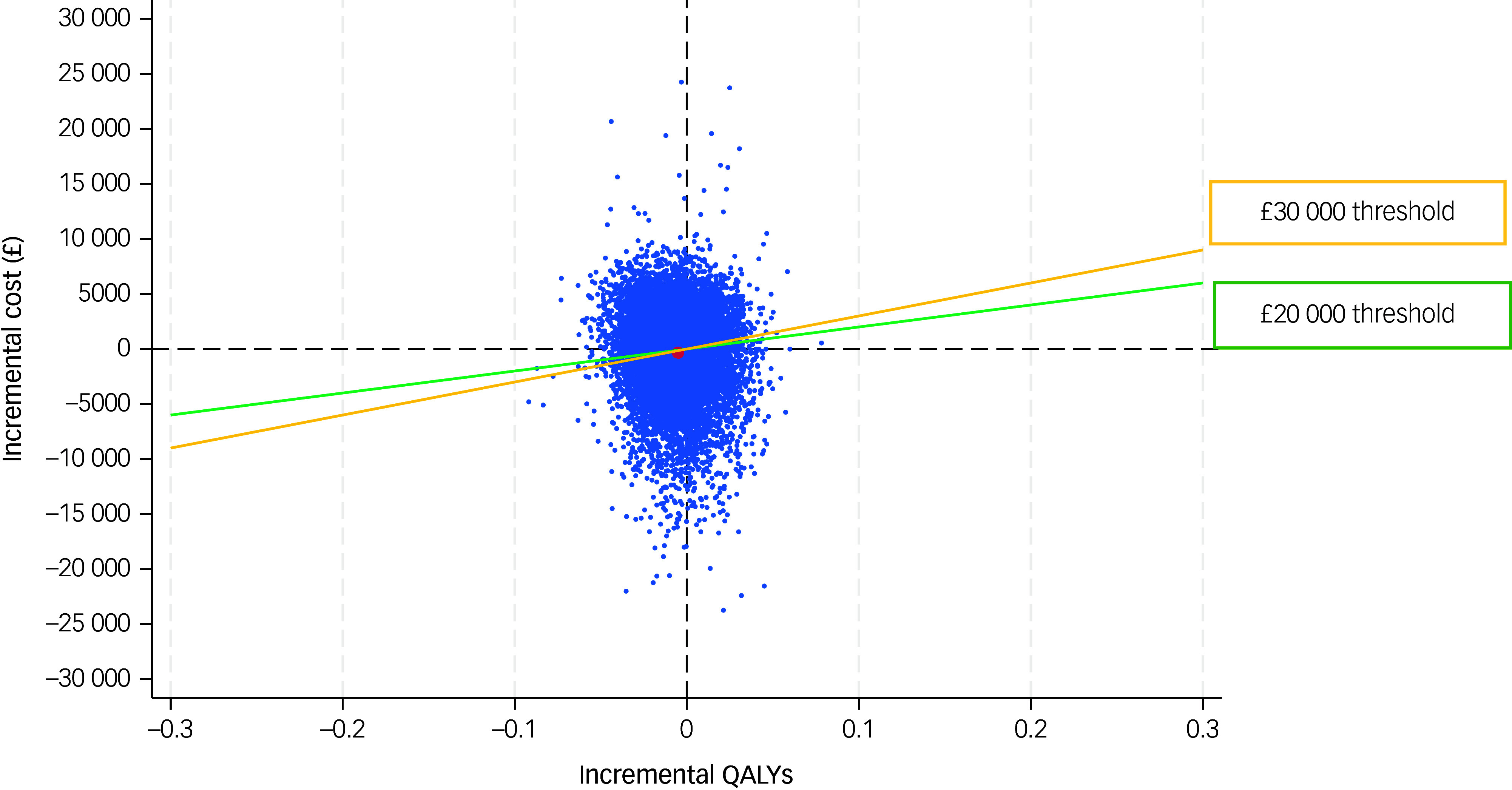
Cost-effectiveness plane of victim improvement package intervention compared with treatment as usual, from a societal cost perspective over 6 months. QALYs, quality-adjusted life years.

The CEP was constructed and is shown in Fig. 1.

**Fig. 2:**
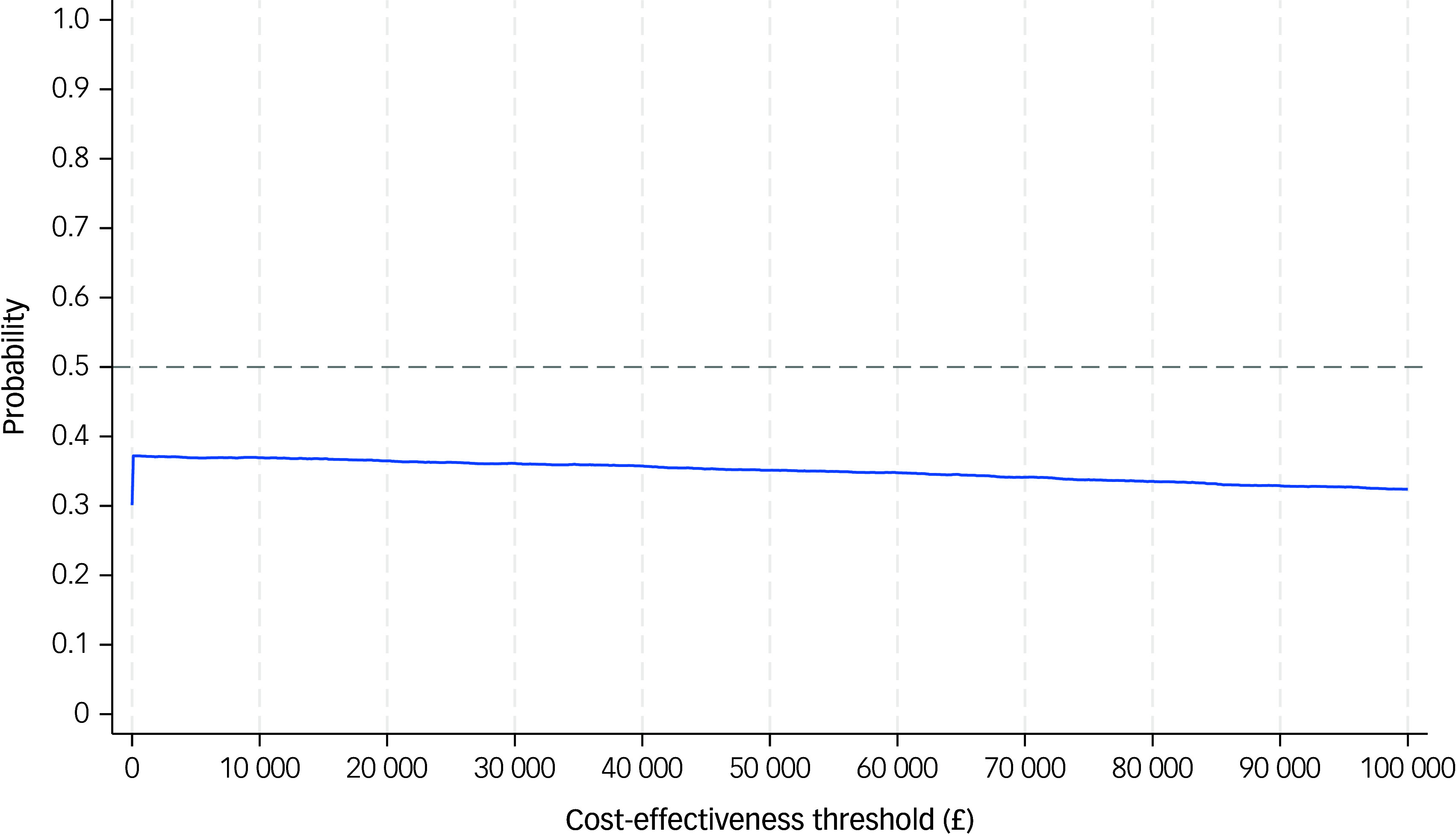
Cost-effectiveness acceptability curve of victim improvement package intervention compared with treatment as usual, from a societal cost perspective over 6 months. The solid line represents the cost-effectiveness acceptability curve (CEAC). The horizontal dashed line on a CEAC graph typically indicates a specific probability threshold, most often the 50% probability level.

The CEAC shows that the probability of VIP being cost-effective relative to TAU was 36%, at a threshold of £20 000 or £30 000 per QALY gained, respectively (Fig. 2).

Normally, as the WTP threshold increases (e.g. from £20 000 to 30 000), the probability that an intervention is cost-effective increases because a higher WTP makes it more acceptable to pay for more expensive interventions with modest effectiveness. In our analysis, the mean difference in QALY was slightly negative (−0.011), indicating worse health outcomes with VIP, and the mean cost difference was also negative (−£881), suggesting lower costs. That places many bootstrap samples in the south-west quadrant of the CEP, where VIP is cheaper but also less effective than TAU. In this quadrant, VIP can be considered cost-effective only if decision-makers are willing to accept some loss in health for a cost saving, which is not standard NICE guidance. As a result, increasing the WTP threshold does not change the judgement, because we are still trading off worse outcomes for lower costs. Hence, even when WTP increases from £20 000 to 30 000 per QALY, the intervention is still not offering any gain in health. Therefore, a cautious interpretation of the results would be that the cost-utility analysis indicates that VIP is slightly less costly and slightly less effective than TAU, but with wide uncertainty around both estimates. While this places VIP in the south-west quadrant of the CEP, the magnitude and significance of the QALY difference do not justify declaring VIP cost-effective or TAU not cost-effective.

### Sensitivity analysis

Results of per-protocol analysis showed no significant differences in resource use (Supplementary Table 7), but a significant negative difference in the costs of both social and legal advice services (mean difference −£487.59 (95% CI: −£908.26 to −£66.91), *P* = 0.023) and informal care (mean difference −£7919.60 (95% CI: −£15 329 to −£509.93), *P* = 0.036) for the VIP arm (Supplementary Table 8). There were no statistically significant differences in utility values and QALYs (mean difference −0.027 (95% CI: −0.068 to 0.014), *P* = 0.204) (Supplementary Table 9). Results for the per-protocol population show lower mean point estimates for cost and QALYs at greater magnitudes compared with the ITT population. The probability of the cost-effectiveness of VIP intervention was also similarly low (33% at a WTP of £20 000 and 32% at a WTP of £30 000; Supplementary Table 10).

Loss to follow-up presented particular challenges. To investigate potential bias from this missingness, we compared baseline demographic and clinical characteristics between participants with complete and incomplete data. No significant differences were observed in regard to age, gender, ethnicity, baseline utility scores or CSRI responses (Supplementary Table 3). Incremental costs and QALYs were also robust when using multiple imputation. VIP intervention continued to show a low probability of cost-effectiveness over the common WTP thresholds (48% at WTP of £20 000 and £30 000, respectively; Supplementary Table 10).

## Discussion

This is the first economic evaluation of an intervention designed to reduce symptoms of depression and/or anxiety in older victims of community crime. This cost-effectiveness analysis is important given that it is the first to be conducted alongside a clinical trial in this population; however, the main finding of the cost-effectiveness analysis described here is that there is a low probability that the addition of VIP intervention to TAU is cost-effective.

### Strengths and limitations

A major strength of the study is that it is based on data from a randomised controlled trial and that best practice methods have been used in the economic evaluation. Compared with other disease areas, there are no economic evaluations conducted in older victims of crime, due to the challenges of timely identification and recruitment of victims into research.^3^

In our study, possible explanations as to why there were so few differences in outcomes between study arms may relate to the lack of effect of the intervention (it is possible that the trial did not provide an adequate test of the intervention), methodology (slow recruitment, small sample size) or population (vulnerable older people). More than a third of participants allocated to receive VIP did not receive any intervention due to practical constraints, such as busy clinical service, therapy availability or participant withdrawal.^36^ Service pressures were particularly acute during the trial. There were notable constraints in therapist capacity and delays in intervention delivery. In addition, not all therapists were CBT trained. These issues had material effects on the fidelity of intervention delivery. In some cases, participants waited several weeks before their first session, which may have lessened the potential benefit. These contextual limitations significantly affected the internal validity of the trial and should be explicitly considered when interpreting the findings.

Some findings showed that participants could benefit from a tailored intervention to support them post-crime. Participants in the TAU arm accessed significantly more primary care services for psychological reasons, needed more informal care and accessed more social and legal advice services. This could be because older victims of community crime experience complex emotions soon after the crime. Offering up to 10 sessions over 3 months may lack the intensity of tailoring required to address the complex emotional and psychological needs of older crime victims. However, the potential costs of leaving this population unsupported will increase, especially if they do not receive help in coping with the immediate effects of crime, effects that can worsen over time affecting their health and well-being.

A limitation of the study is that service demands, staff changes within the police service and altered public confidence in the police posed challenges to the capacity of the police to screen potential participants, which potentially compromised recruitment.^36^ Also, recruitment and follow-up for the trial occurred in part during the COVID-19 pandemic, which may have had an impact on the ability to access services and some responses to participant-reported outcomes. This has undoubtedly distorted costs (services suspended or not available, family members not going out to work, remote intervention delivery), and may have distorted outcomes (although presumably affecting both arms).

Loss to follow-up presented particular challenges. We addressed this using recommended statistical methods.^33^ Multiple imputation included baseline variables and predictors of missingness but, given the extent of missing data, this still poses a risk of bias. Sensitivity analyses using imputed data-sets yielded estimates similar to the complete case analysis, strengthening confidence in our findings. Nevertheless, unmeasured confounding may remain and the results should be interpreted with caution.

The EQ-5D-5L was selected for this evaluation because it is the tool recommended by NICE for use in cost-utility analyses due to its comparability across disease areas and availability of UK value sets.^26^ The EQ-5D instrument has been criticised as being unsuitable for older people, because it is not particularly sensitive to symptoms.^37^ However, more recent reviews have concluded that EQ-5D has good feasibility properties in an older population.^38^ Participants in our study were expected to present with anxiety and/or depression and the EQ-5D tool measures health-related quality of life, covering among others the anxiety/depression domain. Future evaluations could consider the inclusion of complementary patient-reported outcome measures.

Our results should be viewed with caution because we are not able to conclude whether they reflect the lack of treatment effect (whether the VIP intervention did not work or whether it was not properly delivered). The implementation barriers probably attenuated the intervention’s potential clinical and economic impact.

### Implications

The results of the cost-effectiveness analysis need to be taken in the context of the results of the analysis of clinical effectiveness. Being more aware of the needs of older people and identifying potential vulnerabilities, the existence of a service support could improve their quality of life. Victims reported psychological distress associated with the crime. We would, however, recommend exercising caution about imputing causality: people with depressive and/or anxiety symptoms may be more likely to attribute their symptoms to the crime, or those with a history of anxiety and/or depression are more vulnerable to the impact of crime. Therefore, there is a requirement that services provide tailored interventions and address the needs of this group of vulnerable people, which would then make them feel that they would benefit from additional support and communication in the event of experiencing a crime. Such services require effective, easily implemented and cost-effective psychological treatments for depression and anxiety that can be delivered, eventually, by less specialised health workers trained in CBT. Much depends on the way programmes are implemented outside clinical trials, and the extent to which older victims of crime utilise and experience long-term benefits. Decisions about resource allocation should be based on the relative benefits and costs of interventions, although these cannot be the sole criteria used. Further research should consider more robust recruitment strategies that extend beyond police referrals and include community organisations, and also incorporate flexible follow-up options. Ensuring intervention fidelity will be critical, potentially through a centralised therapist training and certification programme. Process evaluations embedded within trials would help in understanding delivery challenges and enhance the sustainability of interventions.

Based on the results of this study, a VIP intervention would not be recommended on the grounds of cost-effectiveness but needs to be interpreted within the wider context of the potential lack of treatment effect, or older people’s preferences and clinical findings. Future research is needed to identify the most cost-effective intervention, including its length and intensity and long-term sustainability.

## Supporting information

Panca et al. supplementary material Panca et al. supplementary material

## Data Availability

Data supporting the findings can be obtained on reasonable request from the corresponding author, M.P. Requests for access to the VIP data will be reviewed on an individual basis by the chief investigator.

## Supplementary material

The supplementary material is available online at https://doi.org/10.1192/bjo.2025.10937
