# Supplementary material for: Cost-effectiveness of a victim improvement package: randomised controlled trial for reduction of continued symptoms of depression or anxiety in older victims of community crime
Source: BJPsych Open. 2026 Jan 7;12(1):e29. doi: 10.1192/bjo.2025.10937 (PMC12835716; doi:10.1192/bjo.2025.10937)
Supplement: Panca et al. supplementary material [file S205647242510937Xsup001.docx]

**Table S1. Resource use unit cost**

|  | **Unit cost (2021/2022) (£)** | **Notes** | **Source** |
| --- | --- | --- | --- |
| **Criminal Justice services (contacts)** | | | |
| UK Police force   - Assault - Burglary - Criminal damage - Fraud - Harassment - Robbery - Theft | £930.66  £608.95  £172.34  £68.94  £930.66  £1160.45  £45.96 | Annual inflator to uprate the costs to 2021/2022 values (The Personal Social Services (PSS) Pay & Prices Index) | Home Office-The economic and social costs of Crime; Second edition 2018 (Average police costs associated with different crimes) |
| Family Liaison Officer | £12.31 | Average family liaison officer salary in the United Kingdom is £23,997 per year. | www.talent.com |
| Victim Support   - Assault - Burglary - Criminal damage - Fraud - Harassment - Robbery - Theft | £723.84  £815.76  £264.26  £114.90  £723.84  £2183.02  £1287.24 | Annual inflator to uprate the costs to 2021/2022 values (The Personal Social Services (PSS) Pay & Prices Index) | Home Office-The economic and social costs of Crime; Second edition 2018 (Average costs of crimes to the Criminal Justice System; Non-legal aid defence) |
| Legal Services (e.g., solicitor)   - Assault - Burglary - Criminal damage - Fraud - Harassment - Robbery - Theft | £ 195.32  £ 45.96  £ 22.98  £ 22.98  £ 195.32  £ 218.30  £ 11.49 | Annual inflator to uprate the costs to 2021/2022 values (The Personal Social Services (PSS) Pay & Prices Index) | Home Office-The economic and social costs of Crime; Second edition 2018 (Average costs of crimes to the Criminal Justice System; Legal Aid) |
| **Social and legal advice services (contacts)** | | | |
| Social worker or care manager | £12 | £42 per hour of patient-related work (excluding qualifications); based on the assumption of a 30-minute contact | Curtis, L. & Burns, A. (2022) Unit Costs of Health and Social Care 2022, Personal Social Services Research Unit, University of Kent, Canterbury. (pg.77) |
| Housing Association/other housing support | £47 | Occupational therapist | Curtis, L. & Burns, A. (2022) Unit Costs of Health and Social Care 2022, Personal Social Services Research Unit, University of Kent, Canterbury (pg.79) |
| Citizen’s Advice Bureau/other advice agency | £47 | Occupational therapist | Curtis, L. & Burns, A. (2022) Unit Costs of Health and Social Care 2022, Personal Social Services Research Unit, University of Kent, Canterbury (pg.79) |
| Samaritans/other advice line | £5.28 | Based on £3.88 cost per contact in 2010–11. Annual inflator to uprate the costs to 2021/2022 values (The Personal Social Services (PSS) Pay & Prices Index) | Curtis, L. & Burns, A. (2011) Unit Costs of Health and Social Care 2011, Personal Social Services Research Unit, University of Kent, Canterbury |
| Mind/other charity service | £104 | Based on £ 88 per hour of direct contact in 2016–17. Annual inflator to uprate the costs to 2021/2022 values (The Personal Social Services (PSS) Pay & Prices Index) | Curtis, L. & Burns, A. (2017) Unit Costs of Health and Social Care 2017, Personal Social Services Research Unit, University of Kent, Canterbury |
| **Healthcare services (contacts)** | | | |
| Hospital Inpatient (days) | £1,378 | Annual inflator to uprate the costs to 2021/2022 values (The Personal Social Services (PSS) Pay & Prices Index) | National Schedule of NHS Costs - Year 2020-21, Non-Elective Inpatients (trim point floor of five days -https://www.england.nhs.uk/wp-content/uploads/2020/11/21-22NT_Annex-B-Guidance-on-currencies.pdf |
| Hospital Outpatient | £235 | Outpatient attendances; Weighted average of all outpatient attendances | Curtis, L. & Burns, A. (2022) Unit Costs of Health and Social Care 2022, Personal Social Services Research Unit, University of Kent, Canterbury |
| Accident & Emergency | £170 | Annual inflator to uprate the costs to 2021/2022 values (The Personal Social Services (PSS) Pay & Prices Index) | National Schedule of NHS Costs - Year 2020-21/ Total Outpatient Attendance / Emergency Medicine Service |
| NHS walk-in-centre | £86 | Lowest level of investigation and treatment | https://www.kingsfund.org.uk/audio-video/key-facts-figures-nhs |
| Paramedics/ambulance | £357.40 | Annual inflator to uprate the costs to 2021/2022 values (The Personal Social Services (PSS) Pay & Prices Index) | National Schedule of NHS Costs - Year 2020-21/Ambulance/ See & Convey |
| Day centre | £58 | Per client attendance; Local authority own-provision social services day care for adults requiring mental health support (age 18-64) | Curtis, L. & Burns, A. (2022) Unit Costs of Health and Social Care 2022, Personal Social Services Research Unit, University of Kent, Canterbury |
| Rehabilitation centre | £178.40 | Assessment for Rehabilitation, Multidisciplinary, Specialist | National Schedule of NHS Costs - Year 2021-22/ Rehabilitation |
| **Primary care and community services (contacts)** | | | |
| General Practitioner (GP) |  |  |  |
| - at home | £236 | Per contact. Annual inflator to uprate the costs to 2021/2022 values (The Personal Social Services (PSS) Pay & Prices Index) | Curtis, L. & Burns, A. (2015) Unit Costs of Health and Social Care 2015, Personal Social Services Research Unit, University of Kent, Canterbury |
| - at clinic | £38 | Per surgery consultation lasting 9.22 minutes | Curtis, L. & Burns, A. (2022) Unit Costs of Health and Social Care 2022, Personal Social Services Research Unit, University of Kent, Canterbury |
| - by phone | £9 | Assumed 5 minutes phone consultation | Curtis, L. & Burns, A. (2022) Unit Costs of Health and Social Care 2022, Personal Social Services Research Unit, University of Kent, Canterbury |
| Practice Nurse |  |  |  |
| - at home | £22 | Assumed equivalent to a home visit of 25 minutes. | Curtis, L. & Burns, A. (2022) Unit Costs of Health and Social Care 2022, Personal Social Services Research Unit, University of Kent, Canterbury |
| - at clinic | £13 | Assumed 15 minutes consultation | Curtis, L. & Burns, A. (2022) Unit Costs of Health and Social Care 2022, Personal Social Services Research Unit, University of Kent, Canterbury |
| - by phone | £9 | Assumed 5 minutes phone consultation | Curtis, L. & Burns, A. (2022) Unit Costs of Health and Social Care 2022, Personal Social Services Research Unit, University of Kent, Canterbury |
| District Nurse/ Specialist Nurse/ Community psychiatric/ mental health nurse |  |  |  |
| - at home | £35 | Assumed equivalent to a home visit of 25 minutes. Annual inflator to uprate the costs to 2021/2022 values (The Personal Social Services (PSS) Pay & Prices Index) | Curtis, L. & Burns, A. (2018) Unit Costs of Health and Social Care 2018, Personal Social Services Research Unit, University of Kent, Canterbury |
| - at clinic | £22 | Assumed 30 minutes consultation; Annual inflator to uprate the costs to 2021/2022 values (The Personal Social Services (PSS) Pay & Prices Index) | Curtis, L. & Burns, A. (2018) Unit Costs of Health and Social Care 2018, Personal Social Services Research Unit, University of Kent, Canterbury |
| - by phone | £7 | Assumed 10 minutes consultation | Curtis, L. & Burns, A. (2022) Unit Costs of Health and Social Care 2022, Personal Social Services Research Unit, University of Kent, Canterbury |
| Occupational therapist/ Counsellor |  |  |  |
| - at home | £21 | Assumed equivalent to a home visit of 25 minutes | Curtis, L. & Burns, A. (2022) Unit Costs of Health and Social Care 2022, Personal Social Services Research Unit, University of Kent, Canterbury |
| - at clinic | £47 | Occupational therapist | Curtis, L. & Burns, A. (2022) Unit Costs of Health and Social Care 2022, Personal Social Services Research Unit, University of Kent, Canterbury |
| - by phone | £8 | Assumed 10 minutes phone consultation. | Curtis, L. & Burns, A. (2022) Unit Costs of Health and Social Care 2022, Personal Social Services Research Unit, University of Kent, Canterbury |
| Non-trial psychology services (individual) | £118 | Per 55 minutes session. Annual inflator to uprate the costs to 2021/2022 values (The Personal Social Services (PSS) Pay & Prices Index) | Curtis, L. & Burns, A. (2017) Unit Costs of Health and Social Care 2017, Personal Social Services Research Unit, University of Kent, Canterbury |
| Non-trial psychology services (group) | £18 | Per service user, therapy sessions lasted 2 hours. Annual inflator to uprate the costs to 2021/2022 values (The Personal Social Services (PSS) Pay & Prices Index) | Curtis, L. & Burns, A. (2017) Unit Costs of Health and Social Care 2017, Personal Social Services Research Unit, University of Kent, Canterbury |
| **Support provided by unpaid (informal) carers** | | | |
| Per hour | £14.77 | Median hourly earnings of a carer in paid employment (year 2022) | https://www.ons.gov.uk/ |

**Table S2. Medication unit cost**

|  | **Dose** | **Unit** | **Drug tariff (November 2022)** |
| --- | --- | --- | --- |
| Amitriptyline hydrochloride (AAH Pharmaceuticals Ltd) | 10 mg | 28 tablets | £0.73 |
| Citalopram (AAH Pharmaceuticals Ltd) | 10 mg | 28 tablets | £1.00 |
| Citalopram (AAH Pharmaceuticals Ltd) | 30 mg | 28 tablets | £1.36 |
| Clomipramine hydrochloride (AAH Pharmaceuticals Ltd) | 200 mg | 28 capsules | £3.44 |
| Diazepam (AAH Pharmaceuticals Ltd) | 2 mg | 28 tablets | £0.79 |
| Diazepam (AAH Pharmaceuticals Ltd) | 5 mg | 28 tablets | £0.81 |
| Diphenhydramine | 50 mg | 20 tablets | £4.46 |
| Fluoxetine hydrochloride (AAH Pharmaceuticals Ltd) | 20 mg | 30 capsules | £1.24 |
| Fluoxetine hydrochloride (AAH Pharmaceuticals Ltd) | 40 mg | 30 capsules | £2.50 |
| Melatonin (AAH Pharmaceuticals Ltd/Flynn Pharma Ltd) | 10 mg | 30 tablets | £6.53 |
| Mirtazapine (AAH Pharmaceuticals Ltd) | 15 mg | 28 tablets | £1.01 |
| Mirtazapine (AAH Pharmaceuticals Ltd) | 30 mg | 28 tablets | £0.99 |
| Mirtazapine (AAH Pharmaceuticals Ltd) | 45 mg | 28 tablets | £1.24 |
| Paroxetine hydrochloride (AAH Pharmaceuticals Ltd) | 20 mg | 30 tablets | £1.62 |
| Paroxetine hydrochloride (AAH Pharmaceuticals Ltd) | 30 mg | 30 tablets | £1.62 |
| Pregabalin (AAH Pharmaceuticals Ltd) | 75 mg | 56 tablets | £4.79 |
| Quetiapine fumarate (AAH Pharmaceuticals Ltd) | 25 mg | 60 tablets | £1.23 |
| Quetiapine fumarate (Accord-UK Ltd) | 50 mg | 60 tablets | £67.66 |
| Risperidone (AAH Pharmaceuticals Ltd) | 3 mg | 60 tablets | £1.93 |
| Sertraline hydrochloride (AAH Pharmaceuticals Ltd) | 50 mg | 28 tablets | £1.04 |
| Sertraline hydrochloride (AAH Pharmaceuticals Ltd) | 100 mg | 28 tablets | £1.22 |
| Sertraline hydrochloride (AAH Pharmaceuticals Ltd) | 200 mg | 28 tablets | £18.00 |
| Simvastatin (AAH Pharmaceuticals Ltd) | 20 mg | 28 tablets | £0.77 |
| Sodium valproate (AAH Pharmaceuticals Ltd) | 500 mg | 100 tablets | £18.35 |
| Temazepam (AAH Pharmaceuticals Ltd) | 10 mg | 28 tablets | £23.61 |
| Temazepam (AAH Pharmaceuticals Ltd) | 20 mg | 28 tablets | £24.25 |
| Venlafaxine hydrochloride (AAH Pharmaceuticals Ltd) | 75 mg | 56 tablets | £10.46 |
| Venlafaxine hydrochloride (Alliance Healthcare (Distribution) Ltd ) | 150 mg | 28 tablets | £18.70 |
| Zopiclone (AAH Pharmaceuticals Ltd) | 3.75 mg | 28 tablets | £1.45 |
| Zopiclone (AAH Pharmaceuticals Ltd) | 7.5 mg | 28 tablets | £1.33 |

**Table S3. Baseline characteristics of participants**

|  | **Randomised to VIP and TAU** | |  | **With complete/incomplete data** | |
| --- | --- | --- | --- | --- | --- |
|  | **VIP**  **(N=65)** | **TAU**  **(N=66)** |  | **Missing data (N=54)** | **No missing data (N=57)** |
|  | **Mean (SD) or n (%)** | **Mean (SD) or n (%)** |  | **Mean (SD) or n (%)** | **Mean (SD) or n (%)** |
| **Age (years)** | 72.1 (5.9) | 72.1 (11.2) |  | 72.5 (5.2) | 72.4 (7.0) |
| **Gender** |  |  |  |  |  |
| Female | 46 (70.8%) | 42 (63.6%) |  | 35 (64.8%) | 40 (70.2%) |
| Male | 19 (29.2%) | 24 (36.6%) |  | 19 (35.2%) | 17 (29.8%) |
| **Ethnicity** |  |  |  |  |  |
| White | 45 (69.2%) | 45 (68.2%) |  | 35 (64.8%) | 42 (73.7%) |
| Black | 5 (7.7%) | 4 (6.1%) |  | 4 (7.4%) | 3 (5.3%) |
| Asian | 8 (12.3%) | 10 (15.2%) |  | 8 (14.8%) | 9 (15.8%) |
| Other | 7 (10.8%) | 7 (10.6%) |  | 7 (13.0%) | 3 (5.2%) |
| **Victim vulnerability** |  |  |  |  |  |
| Recorded vulnerability | 7 (10.8%) | 13 (19.7%) |  | 11 (20.4%) | 7 (12.3%) |
| No recorded vulnerability | 58 (89.2%) | 53 (80.3%) |  | 43 (79.6%) | 50 (87.7%) |
| **Marital status** |  |  |  |  |  |
| Single | 12 (18.5%) | 11 (16.7%) |  | 7 (12.0%) | 14 (24.6%) |
| Married/Cohabitating | 29 (44.6%) | 23 (34.8%) |  | 22 (40.7%) | 22 (38.5%) |
| Widow/Widower | 10 (15.4%) | 19 (23.8%) |  | 12 (22.2%) | 12 (21.1%) |
| Divorced/Separated | 13 (20.0%) | 11 (16.7%) |  | 12 (22.2%) | 7 (12.3%) |
| Other | 1 (1.5%) | 2 (3.0%) |  | 1 (1.9%) | 2 (3.5%) |
| **Education** |  |  |  |  |  |
| Primary | 3 (4.9%) | 3 (5.0%) |  | 3 (6.0%) | 3 (5.8%) |
| Secondary | 34 (55.7%) | 27 (45.0%) |  | 25 (50.0%) | 23 (44.2%) |
| Higher | 24 (39.3%) | 30 (50.0%) |  | 22 (44.0%) | 26 (50.0%) |
| **Living arrangement** |  |  |  |  |  |
| Rented | 27 (41.5%) | 29 (46.0%) |  | 27 (50.0%) | 20 (35.1%) |
| Owner/ Occupier | 36 (55.4%) | 35 (53.0%) |  | 26 (48.2%) | 35 (61.4%) |
| Other | 2 (3.1%) | 2 (3.0%) |  | 1 (1.8%) | 2 (3.5%) |
| **Crime group** |  |  |  |  |  |
| Assault | 5 (7.8%) | 6 (9.1%) |  | 7 (13.2%) | 3 (5.3%) |
| Burglary | 18 (28.1%) | 23 (34.9%) |  | 18 (34.0%) | 17 (29.8%) |
| Criminal damage | 8 (12.5%) | 13 (19.7%) |  | 9 (17.0%) | 10 (17.5%) |
| Fraud | 2 (3.1%) | 1 (1.5%) |  | 0 (0.0%) | 2 (3.5%) |
| Harassment | 4 (6.3%) | 3 (4.6%) |  | 0 (0.0%) | 4 (7.0%) |
| Robbery | 2 (3.1%) | 3 (4.6%) |  | 4 (7.5%) | 1 (1.8%) |
| Theft | 25 (39.1%) | 17 (25.8%) |  | 15 (28.3%) | 20 (35.1%) |
| **Anyone arrested?** |  |  |  |  |  |
| Yes | 7 (10.8%) | 3 (4.6%) |  | 5 (9.4%) | 4 (7.0%) |
| No | 58 (89.2%) | 62 (95.4%) |  | 48 (90.6%) | 53 (93.0%) |
| **Affected daily life** |  |  |  |  |  |
| Yes | 56 (87.5%) | 62 (95.4%) |  | 49 (92.5%) | 50 (89.3%) |
| No | 8 (12.5%) | 3 (4.6%) |  | 4 (7.5%) | 6 (10.7%) |
| **Previously suffered from depression or anxiety?** |  |  |  |  |  |
| Yes | 29 (46.8%) | 40 (61.5%) |  | 28 (53.9%) | 27 (48.2%) |
| No | 33 (53.2%) | 25 (38.5%) |  | 24 (46.1%) | 29 (51.8%) |
| **Sense of safety before crime** |  |  |  |  |  |
| Very safe | 13 (20.0%) | 19 (28.8%) |  | 14 (25.9%) | 15 (26.3%) |
| Safe | 38 (58.5%) | 28 (42.4%) |  | 28 (51.9%) | 23 (40.4%) |
| Neither safe nor unsafe | 7 (10.8%) | 8 (12.1%) |  | 5 (9.3%) | 9 (15.8%) |
| Unsafe | 6 (9.2%) | 10 (15.2%) |  | 6 (11.1%) | 9 (15.8%) |
| Very unsafe | 1 (1.5%) | 1 (1.5%) |  | 1 (1.8%) | 1 (1.7%) |
| **Sense of safety after crime** |  |  |  |  |  |
| Very safe | 0 (0.0) | 0 (0.0%) |  | 0 (0.0%) | 0 (0.0%) |
| Safe | 9 (13.9%) | 4 (6.1%) |  | 5 (9.3%) | 5 (8.8%) |
| Neither safe nor unsafe | 7 (10.8%) | 11 (16.7%) |  | 7 (13.0%) | 7 (12.2%) |
| Unsafe | 31 (47.7%) | 33 (50.0%) |  | 23 (42.6%) | 31 (54.4%) |
| Very unsafe | 18 (27.7%) | 18 (27.3%) |  | 19 (35.2%) | 14 (24.6%) |
| **Severity of anxiety symptom (GAD-2)** | 6.4 (1.7) | 6.6 (1.6) |  | 6.8 (1.5) | 6.1 (1.7) |
| **Severity of depression symptoms (PHQ-2)** | 5.4 (1.9) | 5.7 (1.9) |  | 5.8 (1.9) | 5.1 (1.9) |
| **Utility values (EQ-5D-5L)** |  |  |  | 0.400 (0.331) | 0.505 (0.319) |
| **Resource use** |  |  |  |  |  |
| Criminal Justice |  |  |  | 3.1 (3.5) | 4.2 (7.0) |
| Social Services |  |  |  | 5.2 (17.8) | 5.1 (13.1) |
| Inpatient |  |  |  | 0.7 (4.1) | 0.9 (4.4) |
| Outpatient |  |  |  | 2.6 (4.6) | 2.9 (5.1) |
| General Practitioner (Crime Reason) |  |  |  | 0.7 (1.9) | 0.3 (1.3) |
| Community Nurse |  |  |  | 2.0 (7.4) | 1.1 (3.8) |
| Other Psychological Services |  |  |  | 0.2 (0.8) | 0.2 (0.8) |

*SD standard deviation; GAD-2 Generalized Anxiety Disorder 2 item; PHQ-2 Patient Health Questionnaire-2

**TableS4. Mean resource use at baseline and follow-up time points per participant and baseline-adjusted mean difference between trial arms over 6 months trial duration**

|  | **VIP (N=65)** | | **TAU (N=66)** | | **VIP vs TAU** |  |
| --- | --- | --- | --- | --- | --- | --- |
|  | **N (n)** | **Mean (SD)** | **N (n)** | **Mean (SD)** | **Adjusted mean difference (95% CI)*** | **P value** |
| 1. **Criminal Justice services** | | | | | | |
| Baseline (3 months) | 53 (41) | 4.15 (3.49) | 58 (46) | 5.28 (7.41) |  |  |
| 6 months follow-up | 38 (12) | 2.58 (1.62) | 45 (15) | 5.87 (7.01) |  |  |
| 9 months follow-up | 37 (6) | 6.17 (4.75) | 37 (12) | 2.42 (1.38) |  |  |
| Over 6 months | 62 (43) | 5.53 (5.74) | 63 (50) | 7.20 (11.89) | -0.14 (-1.41 to 1.13) | 0.829 |
| 1. **Social and legal advice services** | | | | | | |
| Baseline (3 months) | 53 (20) | 15.95 (23.81) | 57 (15) | 15.8 (24.78) |  |  |
| 6 months follow-up | 31 (8) | 9.63 (3.62) | 39 (8) | 31.75 (65.17) |  |  |
| 9 months follow-up | 36 (15) | 8.93 (7.68) | 36 (15) | 21.33 (41.11) |  |  |
| Over 6 months | 59 (27) | 19.63 (26.09) | 62 (25) | 32.44 (81.01) | -5.56 (-21.21 to 10.09) | 0.486 |
| 1. **Healthcare services** | | | | | | |
| **Inpatient services** | | | | | | |
| Baseline (3 months) | 54 (6) | 13.67 (13.43) | 58 (4) | 2.50 (1.91) |  |  |
| 6 months follow-up | 37 (5) | 7.60 (9.37) | 45 (1) | 4. 00 (0.00) |  |  |
| 9 months follow-up | 36 (3) | 8.33 (2.08) | 37 (1) | 1.00 (0.00) |  |  |
| Over 6 months | 62 (12) | 12.08 (13.44) | 63 (6) | 2.50 (1.76) | 0.41 (-0.27 to 1.08) | 0.236 |
| **Outpatient services** | | | | | | |
| Baseline (3 months) | 54 (36) | 5.61 (7.22) | 59 (33) | 3.33 (3.17) |  |  |
| 6 months follow-up | 38 (26) | 4.04 (3.96) | 45 (26) | 3.46 (2.87) |  |  |
| 9 months follow-up | 34 (22) | 5.45 (6.92) | 34 (23) | 2.83 (2.44) |  |  |
| Over 6 months | 60 (46) | 9.28 (13.81) | 62 (49) | 5.41 (4.32) | 0.50 (-1.36 to 2.37) | 0.599 |
| **Primary care services** | | | | | | |
| **Distress associated with crime** | | | | | | |
| Baseline | 55 (10) | 1.30 (0.48) | 59 (14) | 3.14 (3.42) |  |  |
| 3 months follow-up | 38 (1) | 1.00 (0.00) | 45 (4) | 1.50 (0.58) |  |  |
| 6 months follow-up | 37 (0) | 0.00 (0.00) | 37 (2) | 2.00 (0.00) |  |  |
| Over 6 months | 62 (10) | 1.40 (0.52) | 63 (15) | 3.60 (3.31) | -0.12 (-0.34 to 0.09) | 0.265 |
| **Psychological reason** | | | | | | |
| Baseline (3 months) | 55 (4) | 2.5 (1.73) | 59 (7) | 4.00 (5.07) |  |  |
| 6 months follow-up | 38 (4) | 1.25 (0.50) | 45 (8) | 1.75 (0.89) |  |  |
| 9 months follow-up | 37 (1) | 1.00 (0.00) | 37 (5) | 1.60 (0.89) |  |  |
| Over 6 months | 61 (8) | 2.00 (1.31) | 63 (17) | 2.94 (3.77) | -0.25 (-0.48 to -0.02) | **0.032** |
| **Physical reason** | | | | | | |
| Baseline (3 months) | 54 (42) | 3.67 (6.11) | 58 (39) | 2.74 (2.67) |  |  |
| 6 months follow-up | 38 (25) | 1.92 (1.22) | 45 (34) | 3.09 (4.07) |  |  |
| 9 months follow-up | 36 (25) | 2.48 (2.00) | 35 (29) | 2.21 (1.42) |  |  |
| Over 6 months | 61 (53) | 4.98 (6.18) | 62 (54) | 5.11 (4.62) | -0.89 (-2.07 to 0.29) | 0.141 |
| **Community nurses** | | | | | | |
| Baseline (3 months) | 54 (13) | 9.38 (13.08) | 59 (13) | 4.08 (6.46) |  |  |
| 6 months follow-up | 38 (9) | 4.67 (5.72) | 45 (11) | 2.36 (2.66) |  |  |
| 9 months follow-up | 36 (11) | 3.00 (3.35) | 36 (10) | 3.60 (4.55) |  |  |
| Over 6 months | 62 (21) | 9.38 (11.97) | 63 (22) | 5.23 (8.56) | 0.40 (-0.64 to 1.45) | 0.449 |
| **Other psychology/mental services** | | | | | | |
| Baseline (3 months) | 55 (9) | 1.89 (1.27) | 57 (4) | 2.25 (1.89) |  |  |
| 6 months follow-up | 37 (4) | 4.00 (0.82) | 45 (3) | 3.67 (2.08) |  |  |
| 9 months follow-up | 36 (3) | 1.00 (0.00) | 36 (1) | 3.00 (0.00) |  |  |
| Over 6 months | 62 (14) | 2.57 (1.91) | 62 (8) | 2.88 (1.81) | 0.12 (-0.28 to 0.51) | 0.569 |
| 1. **Unpaid/informal care (hours)** | | | | | | |
| Baseline (3 months) | 56 (14) | 39.86 (33.68) | 59 (14) | 25.79 (47.88) |  |  |
| 6 months follow-up | 38 (6) | 21.50 (8.38) | 44 (10) | 30.30 (50.06) |  |  |
| 9 months follow-up | 37 (12) | 12.71 (12.82) | 37 (6) | 49.67 (60.26) |  |  |
| Over 6 months | 62 (17) | 49.38 (37.95) | 63 (17) | 56.59 (110.77) | -8.92 (-19.46 to 1.62) | 0.097 |
| 1. **State benefits** | | | | | | |
| Baseline (3 months) | 56 (39) | 1.74 (0.68) | 58 (38) | 1.74 (0.76) |  |  |
| 6 months follow-up | 37 (18) | 1.50 (0.62) | 44 (32) | 1.53 (0.62) |  |  |
| 9 months follow-up | 37 (17) | 1.53 (0.80) | 36 (22) | 1.41 (0.73) |  |  |
| Over 6 months | 62 (50) | 2.42 (1.75) | 63 (54) | 2.70 (1.79) | -0.40 (-0.89 to 0.09) | 0.112 |
| 1. **Prescriptions** | | | | | | |
| Baseline (3 months) | 51 (12) | 1.50 (0.80) | 56 (17) | 1.24 (0.56) |  |  |
| 6 months follow-up | 37 (6) | 1.33 (0.52) | 45 (13) | 1.08 (0.28) |  |  |
| 9 months follow-up | 34 (7) | 1.29 (0.49) | 39 (13) | 1.08 (0.28) |  |  |
| Over 6 months | 60 (15) | 2.33 (1.63) | 63 (21) | 2.33 (1.32) | -0.20 (-0.50 to 0.10) | 0.192 |

*Baseline-adjusted differences calculated using bias-corrected and accelerated bootstrapped regression

**TableS5.** **Mean service costs (£) at baseline and follow-up time points per participant and baseline-adjusted mean difference between trial arms over 6 months trial duration**

|  | **VIP (N=65)** | | **TAU (N=66)** | | **VIP vs TAU** |  |
| --- | --- | --- | --- | --- | --- | --- |
|  | **N (n)** | **Mean (SD)** | **N (n)** | **Mean (SD)** | **Adjusted mean difference**  **(95% CI)*** | **P value** |
| **Criminal Justice services** | | | | | | |
| Baseline (3 months) | 53 (41) | 1,458.78 (2,329.62) | 58 (46) | 1,865.69 (3,692.85) |  |  |
| 6 months follow-up | 38 (11) | 520.24 (629.39) | 45 (15) | 1,512.95 (1,342.10) |  |  |
| 9 months follow-up | 37 (6) | 407.88 (293.26) | 37 (12) | 893.52 (964.12) |  |  |
| Over 6 months | 30 (8) | 548.73 (867.90) | 32 (15) | 1,972.70 (1,652.75) | -325.40 (-823.15 to 172.34) | 0.200 |
| **Social and legal advice services** | | | | | |  |
| Baseline (3 months) | 56 (11) | 259.57 (405.51) | 58 (11) | 246.35 (359.64) |  |  |
| 6 months follow-up | 34 (2) | 280.00 (192.33) | 41 (3) | 1,491.67 (2,371.54) |  |  |
| 9 months follow-up | 36 (6) | 94.09 (69.44) | 37 (10) | 832.10 (1,018.91) |  |  |
| Over 6 months | 26 (4) | 157.75 (179.2) | 29 (8) | 1,574.50 (2,354.13) | -496.81 (-884.19 to -109.44) | **0.012** |
| **Out-of-pocket expenses** | | | | | |  |
| Baseline (3 months) | 55 (10) | 31.50 (8.83) | 57 (7) | 911.00 (1,927.87) |  |  |
| 6 months follow-up | 36 (7) | 26.43 (6.9) | 43 (9) | 146.22 (333.96) |  |  |
| 9 months follow-up | 36 (8) | 27.56 (6.42) | 36 (7) | 170.00 (366.12) |  |  |
| Over 6 months | 27 (8) | 46.93 (18.) | 30 (6) | 393.50 (804.87) | -11.59 (-23.68 to 0.50) | 0.060 |
| **Healthcare services** | | | | | | |
| Baseline (3 months) | 48 (42) | 3,579.32 (10,228.85) | 55 (47) | 979.52 (1,665.83) |  |  |
| 6 months follow-up | 34 (30) | 2450.71 (6,335.72) | 44 (40) | 690.76 (1,127.41) |  |  |
| 9 months follow-up | 32 (27) | 2,117.67 (3,848.12) | 31 (29) | 606.65 (615.99) |  |  |
| Over 6 months | 24 (24) | 3,762.89 (6,239.66) | 27 (27) | 943.12 (933.66) | 2,075.82 (-154.08 to 4,305.71) | 0.068 |
| **Support provided by unpaid (informal) carers** | | | | | |  |
| Baseline (3 months) | 56 (14) | 7,064.28 (5,968.89) | 59 (14) | 4,570.26 (8,486.53) |  |  |
| 6 months follow-up | 38 (6) | 7,621.32 (2,972.14) | 44 (10) | 10,740.74 (17,743.72) |  |  |
| 9 months follow-up | 37 (12) | 4,504.85 (4,546.13) | 37 (6) | 17,605.84 (21,360.37) |  |  |
| Over 6 months | 30 (10) | 8,241.66 (8,302.89) | 32 (6) | 27,649.44 (45,340.2) | -6,643.49 (-13,058.54 to -228.43) | **0.042** |
| **Prescriptions** | | | | | | |
| Baseline (3 months) | 51 (8) | 45.92 (47.73) | 54 (12) | 10.56 (20.83) |  |  |
| 6 months follow-up | 37 (4) | 40.38 (44.84) | 45 (10) | 26.74 (47.47) |  |  |
| 9 months follow-up | 34 (4) | 40.38 (44.84) | 39 (6) | 6.67 (7.23) |  |  |
| Over 6 months | 60 (9) | 76.71 (83.07) | 62 (15) | 28.94 (50.26) | -1.93 (-11.84 to 7.97) | 0.702 |

*Baseline-adjusted differences calculated using bias-corrected and accelerated bootstrapped regressions

**TableS6.** **Mean utility values (derived from responses to the EQ-5D-5L) at baseline and follow-up time points per participant and baseline-adjusted mean difference in QALYs between trial arms over 6 months trial duration**

|  | **VIP** | | **TAU** | | **VIP vs TAU** |  |
| --- | --- | --- | --- | --- | --- | --- |
|  | **Mean** | **(95% CI)** | **Mean** | **(95% CI)** | **Adjusted mean difference (95% CI)*** | **P value** |
| Baseline (3 months) | 0.468  (n=54) | (0.384 to 0.551) | 0.441  (n=57) | (0.349 to 0.534) |  |  |
| 6 months follow-up | 0.536  (n=31) | (0.418 to 0.655) | 0.588  (n=42) | (0.490 to 0.686) |  |  |
| 9 months follow-up | 0.526  (n=32) | (0.424 to 0.628) | 0.483  (n=34) | (0.346 to 0.620) |  |  |
| QALYs | 0.260  (n=26) | (0.203 to 0.318) | 0.288  (n=31) | (0.232 to 0.344) | -0.011 (-0.048 to 0.023) | 0.496 |

CI confidence interval; QALY quality-adjusted life year

**Table S7. Mean resource use at baseline and follow-up time points per participant and baseline-adjusted mean difference between trial arms over 6 months trial duration (per-protocol analyses)**

|  | **VIP (n=42)** | | **TAU (n=66)** | | **VIP vs TAU** | |
| --- | --- | --- | --- | --- | --- | --- |
|  | **N (n)** | **Mean (SD)** | **N (n)** | **Mean (SD)** | **Adjusted mean difference**  **(95% CI)*** | **P value** |
| 1. **Criminal Justice services** | | | | | | |
| Baseline (3 months) | 33 (24) | 3.92 (3.72) | 58 (46) | 5.28 (7.41) |  |  |
| 6 months follow-up | 26 (8) | 2.88 (1.46) | 45 (15) | 5.87 (7.01) |  |  |
| 9 months follow-up | 23 (4) | 7.75 (5.19) | 37 (12) | 2.42 (1.38) |  |  |
| Over 6 months | 39 (26) | 5.69 (6.87) | 63 (50) | 7.20 (11.89) | 0.41 (-1.11 to 1.94) | 0.594 |
| 1. **Social and legal advice services** | | | | | | |
| Baseline (3 months) | 34 (15) | 15.73 (24.08) | 57 (15) | 15.80 (24.78) |  |  |
| 6 months follow-up | 20 (6) | 9.83 (3.82) | 39 (8) | 31.75 (65.17) |  |  |
| 9 months follow-up | 23 (12) | 9.17 (8.22) | 36 (15) | 21.33 (41.11) |  |  |
| Over 6 months | 38 (21) | 19.29 (24.38) | 62 (25) | 32.44 (81.01) | -5.32 (-24.55 to 13.92) | 0.588 |
| 1. **Healthcare services** | | | | | | |
| **Inpatient services** | | | | | | |
| Baseline (3 months) | 35 (3) | 15.67 (13.58) | 58 (4) | 2.50 (1.91) |  |  |
| 6 months follow-up | 25 (4) | 4.25 (6.50) | 45 (1) | 4.00 (0.00) |  |  |
| 9 months follow-up | 23 (2) | 9.50 (0.71) | 37 (1) | 1.00 (0.00) |  |  |
| Over 6 months | 39 (7) | 11.86 (15.06) | 63 (6) | 2.50 (1.76) | 0.49 (-0.21 to 1.19) | 0.169 |
| **Outpatient services** | | | | | | |
| Baseline (3 months) | 35 (24) | 5.08 (5.69) | 59 (33) | 3.33 (3.17) |  |  |
| 6 months follow-up | 26 (18) | 4.22 (4.47) | 45 (26) | 3.46 (2.87) |  |  |
| 9 months follow-up | 23 (15) | 4.53 (4.22) | 34 (23) | 2.83 (2.44) |  |  |
| Over 6 months | 39 (31) | 8.58 (12.15) | 62 (49) | 5.41 (4.32) | 0.51 (-1.30 to 2.31) | 0.583 |
| **Primary care services** | | | | | | |
| **Distress associated with crime** | | | | | | |
| Baseline (3 months) | 35 (5) | 1.20 (0.45) | 59 (14) | 3.14 (3.42) |  |  |
| 6 months follow-up | 26 (1) | 1.00 (0.00) | 45 (4) | 1.50 (0.58) |  |  |
| 9 months follow-up | 23 (0) | 0.00 (0.00) | 37 (2) | 2.00 (0.00) |  |  |
| Over 6 months | 39 (5) | 1.40 (0.55) | 63 (15) | 3.60 (3.31) | -0.12 (-0.38 to 0.14) | 0.355 |
| **Psychological reason** | | | | | | |
| Baseline (3 months) | 36 (3) | 2.67 (2.08) | 59 (7) | 4.00 (5.07) |  |  |
| 6 months follow-up | 26 (3) | 1.00 (0.00) | 45 (8) | 1.75 (0.89) |  |  |
| 9 months follow-up | 23 (1) | 1.00 (0.00) | 37 (5) | 1.60 (0.89) |  |  |
| Over 6 months | 39 (6) | 2.00 (1.55) | 63 (17) | 2.94 (3.77) | -0.23 (-0.56 to 0.09) | 0.162 |
| **Physical reason** | | | | | | |
| Baseline (3 months) | 35 (26) | 2.19 (1.60) | 58 (39) | 2.74 (2.67) |  |  |
| 6 months follow-up | 26 (18) | 2.11 (1.37) | 45 (34) | 3.09 (4.07) |  |  |
| 9 months follow-up | 23 (15) | 2.80 (2.51) | 35 (29) | 2.21 (1.42) |  |  |
| Over 6 months | 39 (33) | 4.15 (4.24) | 62 (54) | 5.11 (4.62) | -0.48 (-1.85 to 0.88) | 0.489 |
| **Community nurses** | | | | | | |
| Baseline (3 months) | 34 (8) | 3.38 (2.83) | 59 (13) | 4.08 (6.46) |  |  |
| 6 months follow-up | 26 (6) | 3.33 (2.25) | 45 (11) | 2.36 (2.66) |  |  |
| 9 months follow-up | 22 (7) | 3.86 (3.98) | 36 (10) | 3.60 (4.55) |  |  |
| Over 6 months | 39 (14) | 5.29 (5.11) | 63 (22) | 5.23 (8.56) | 0.65 (-0.15 to 1.46) | 0.113 |
| **Other psychology/mental services** | | | | | | |
| Baseline (3 months) | 36 (4) | 2.50 (1.73) | 57 (4) | 2.25 (1.89) |  |  |
| 6 months follow-up | 25 (4) | 4.00 (0.82) | 45 (3) | 3.67 (2.08) |  |  |
| 9 months follow-up | 22 (2) | 1.00 (0.00) | 36 (1) | 3.00 (0.00) |  |  |
| Over 6 months | 39 (8) | 3.50 (2.07) | 62 (8) | 2.88 (1.81) | 0.27 (-0.22 to 0.75) | 0.281 |
| 1. **Unpaid/informal care (hours)** | | | | | | |
| Baseline (3 months) | 36 (10) | 39.00 (40.17) | 59 (14) | 25.79 (47.88) |  |  |
| 6 months follow-up | 26 (5) | 18.80 (5.76) | 44 (10) | 30.30 (50.06) |  |  |
| 9 months follow-up | 23 (9) | 8.61 (8.33) | 37 (6) | 49.67 (60.26) |  |  |
| Over 6 months | 39 (13) | 43.19 (39.36) | 63 (17) | 56.59 (110.77) | -9.51 (-22.29 to 3.28) | 0.145 |
| 1. **State benefits** | | | | | | |
| Baseline (3 months) | 36 (23) | 1.70 (0.63) | 58 (38) | 1.74 (0.76) |  |  |
| 6 months follow-up | 25 (13) | 1.54 (0.66) | 44 (32) | 1.53 (0.62) |  |  |
| 9 months follow-up | 23 (12) | 1.58 (0.90) | 36 (22) | 1.41 (0.73) |  |  |
| Over 6 months | 39 (32) | 2.44 (1.90) | 63 (54) | 2.70 (1.79) | -0.17 (-0.75 to 0.41) | 0.561 |
| 1. **Prescriptions** | | | | | | |
| Baseline (3 months) | 33 (7) | 1.29 (0.76) | 56 (17) | 1.24 (0.56) |  |  |
| 6 months follow-up | 25 (3) | 1.33 (0.58) | 45 (13) | 1.08 (0.28) |  |  |
| 9 months follow-up | 21 (4) | 1.25 (0.50) | 39 (13) | 1.08 (0.28) |  |  |
| Over 6 months | 38 (9) | 2.00 (1.41) | 63 (21) | 2.33 (1.32) | -0.17 (-0.50 to 0.17) | 0.323 |

*Baseline-adjusted differences calculated using bias-corrected and accelerated bootstrapped regressions

**Table S8. Mean service costs (£) at baseline and follow-up time points per participant and baseline-adjusted mean difference between trial arms over 6 months trial duration (per-protocol analyses)**

|  | **VIP (n=42)** | | **TAU (n=66)** | | **VIP vs TAU** | |
| --- | --- | --- | --- | --- | --- | --- |
|  | **N (n)** | **Mean (SD)** | **N (n)** | **Mean (SD)** | **Adjusted mean difference**  **(95% CI)*** | **P value** |
| **Criminal Justice services** | | | | | | |
| Baseline (3 months) | 33 (24) | 1,505.17 (2,498.66) | 58 (46) | 1,865.69 (3,692.85) |  |  |
| 6 months follow-up | 26 (8) | 578.89 (694.47) | 45 (15) | 1,512.95 (1,342.10) |  |  |
| 9 months follow-up | 23 (4) | 350.43 (344.24) | 37 (12) | 893.52 (964.12) |  |  |
| Over 6 months | 20 (6) | 639.73 (996.84) | 32 (15) | 1,972.70 (1,652.75) | -273.21 (-849.81 to 303.38) | 0.353 |
| **Social and legal advice services** | | | | | | |
| Baseline (3 months) | 36 (7) | 313.90 (493.56) | 58 (11) | 246.35 (359.64) |  |  |
| 6 months follow-up | 23 (2) | 280.00 (192.33) | 41 (3) | 1,491.67 (2,371.54) |  |  |
| 9 months follow-up | 23 (5) | 84.70 (73.27) | 37 (10) | 832.10 (1018.91) |  |  |
| Over 6 months | 17 (3) | 163.33 (219.04) | 29 (8) | 1,574.50 (2,354.13) | -487.59 (-908.26 to -66.91) | **0.023** |
| **Out-of-pocket expenses** | | | | | | |
| Baseline (3 months) | 35 (7) | 30.71 (10.18) | 57 (7) | 911.00 (1927.87) |  |  |
| 6 months follow-up | 25 (5) | 25.00 (7.91) | 43 (9) | 146.22 (333.96) |  |  |
| 9 months follow-up | 22 (6) | 26.74 (7.39) | 36 (7) | 170.00 (366.12) |  |  |
| Over 6 months | 18 (6) | 42.57 (19.04) | 30 (6) | 393.50 (804.87) | -13.12 (-27.21 to 0.98) | 0.068 |
| **Healthcare services** | | | | | | |
| Baseline (3 months) | 32 (29) | 3,010.54 (8,569.17) | 55 (47) | 979.52 (1,665.83) |  |  |
| 6 months follow-up | 22 (21) | 1,786.20 (4,423.49) | 44 (40) | 690.76 (1,127.41) |  |  |
| 9 months follow-up | 21 (17) | 2,615.48 (4,444.69) | 31 (29) | 606.65 (615.99) |  |  |
| Over 6 months | 16 (16) | 4,741.21 (7,217.85) | 27 (27) | 943.12 (933.66) | 2,546.79 (-47.51 to 5,141.09) | 0.054 |
| **Support provided by unpaid (informal) carers** | | | | | | |
| Baseline (3 months) | 36 (10) | 6,912.36 (7,119.08) | 59 (14) | 4,570.26 (8,486.53) |  |  |
| 6 months follow-up | 26 (5) | 6,664.22 (2,042.49) | 44 (10) | 10,740.74 (17,743.72) |  |  |
| 9 months follow-up | 23 (9) | 3,052.47 (2,952.23) | 37 (6) | 17,605.84 (21,360.37) |  |  |
| Over 6 months | 20 (9) | 6,203.40 (5,551.32) | 32 (6) | 27,649.44 (45,340.20) | -7,919.60 (-15,329.26 to -509.93) | **0.036** |
| **Prescriptions** | | | | | | |
| Baseline (3 months) | 33 (5) | 38.36 (52.89) | 54 (12) | 10.56 (20.83) |  |  |
| 6 months follow-up | 25 (2) | 41.10 (53.88) | 45 (10) | 26.74 (47.47) |  |  |
| 9 months follow-up | 21 (2) | 41.10 (53.88) | 39 (6) | 6.67 (7.23) |  |  |
| Over 6 months | 38 (6) | 59.37 (67.17) | 62 (15) | 28.94 (50.26) | -1.56 (-12.08 to 8.96) | 0.771 |

*Baseline-adjusted differences calculated using bias-corrected and accelerated bootstrapped regressions

**TableS9. Mean utility values (derived from responses to the EQ-5D-5L) at baseline and follow-up time points per participant and baseline-adjusted mean difference in QALYs between trial arms over 6 months trial duration (per-protocol analysis)**

|  | **VIP** | | **TAU** | | **VIP vs TAU** |  |
| --- | --- | --- | --- | --- | --- | --- |
|  | **Mean** | **(95% CI)** | **Mean** | **(95% CI)** | **Adjusted mean difference (95% CI)*** | **P value** |
| Baseline (3 months) | 0.468  (n=35) | (0.357 to 0.578) | 0.441  (n=57) | (0.349 to 0.534) |  |  |
| 6 months follow-up | 0.469  (n=22) | (0.324 to 0.614) | 0.588  (n=42) | (0.490 to 0.686) |  |  |
| 9 months follow-up | 0.481  (n=21) | (0.348 to 0.614) | 0.483  (n=34) | (0.346 to 0.620) |  |  |
| QALY | 0.231  (n=18) | (0.156 to 0.305) | 0.288  (n=31) | (0.232 to 0.344) | -0.027 (-0.068 to 0.014) | 0.204 |

CI confidence interval; QALY quality-adjusted life year

**Table S10. Cost-effectiveness of VIP intervention versus TAU: per-protocol and imputed data analyses**

|  | **Incremental cost**  **(95% CI)** | **QALY gained**  **(95% CI)** | **ICER** | **Probability CE £20,000** | **Probability CE £30,000** |
| --- | --- | --- | --- | --- | --- |
| Per-protocol analysis | -£3,179 (-£9,489 to £3,131) | -0.025 (-0.063 to 0.013) | £127,735 | 33% | 32% |
| Multiple imputation* | -£333 (-£413 to -£253) | -0.005 (-0.005 to 0.005) | £65,324 | 48% | 48% |

* The imputation model includes GAD-2 (Generalized Anxiety Disorder 2 item) and PHQ-2 (Patient Health Questionnaire -2) baseline data, accommodation, age, gender, 6-month follow-up scores, site, and therapist as variables to impute the costs and utilities

ICER incremental cost effectiveness ratio; CI confidence interval; QALY quality-adjusted life year; CE cost-effectiveness
